# Clinical Characteristics and Mortality in Community-Acquired Sepsis and Septic Shock Patients in the Intensive Care Unit Setting in Latvia: An Observational Study

**DOI:** 10.3390/healthcare13182264

**Published:** 2025-09-10

**Authors:** Laura Puceta, Anna Zilde, Girts Freijs, Peteris Oss, Uga Dumpis

**Affiliations:** 1 Faculty of Medicine, University of Latvia, LV-1004 Riga, Latvia; 2 Department of Internal Medicine, Pauls Stradins Clinical University Hospital, LV-1002 Riga, Latvia; 3 Department of Resuscitation and Intensive Care, Pauls Stradins Clinical University Hospital, LV-1002 Riga, Latvia; 4 Department of Infectious Diseases and Hospital Epidemiology, Pauls Stradins Clinical University Hospital, LV-1002 Riga, Latvia

**Keywords:** community acquired, sepsis, ICU, mortality

## Abstract

**Background:** Sepsis and septic shock are medical emergencies with increasing incidence and high intrahospital mortality rates, as they are common causes of admission to the intensive care units (ICU). Early recognition and adequate treatment are important in reducing mortality. Limited data are available on characteristics and outcomes of community-acquired sepsis and septic shock in Latvia. **Methods:** In this single-center cohort study, we explore clinical characteristics and outcomes of 86 community-acquired sepsis and septic shock cases admitted to the ICU of a clinical university hospital between 2014 and 2018. **Results:** The ICU and intrahospital mortality rates were 45% and 58%, respectively. Respiratory tract infections, particularly pneumonia and abdominal and skin/soft tissue infections, were the most common sites of origin. Overall, 95% of patients initially received appropriate antibiotic treatment. However, the median time to antibiotics was 225 min (IQR 118–407 min), and only 10% received antimicrobial treatment within 1 h. **Conclusions:** The number of patients enrolled indicates that sepsis cases are underreported on a national level. The results also demonstrate high mortality rates among ICU patients with community-acquired sepsis and septic shock, compared to other reports. The analysis indicates that early recognition of sepsis signs and organ failure is crucial for improvements in treatment outcomes.

## 1. Introduction

Sepsis is a life-threatening syndrome induced by infection that has an increasingly reported incidence, which is likely associated with an increased recognition, an ageing population, and a greater burden of comorbidities [1,2,3]. Although the true incidence is unknown, estimates indicate that sepsis is a leading cause of mortality and critical illness worldwide [4,5]. The annual incidence of sepsis is variable in different studies, and the most cited are based on the International Classification of Diseases, 9th and 10th Revision codes (ICD-9 and ICD-10) in hospital discharge databases, where the incidence is reported to be between 132 and 300 per 100,000 population [6,7]. ICD code abstractions have been compared to medical record reviews for identification of traditional severe sepsis and have shown good specificity but poor sensitivity; thus, structured manual review of medical records remains a more definitive strategy for identifying community-acquired sepsis cases.

Approximately 70% of sepsis cases are community-acquired; therefore, early recognition and antibacterial treatment are crucial [8]. Common risk factors for sepsis include age (elderly over 65 years and infants being more vulnerable), impaired immune response (e.g., HIV/AIDS, splenectomy, malignancy, diabetes mellitus), pre-existing organ dysfunction (e.g., chronic kidney disease, heart failure), and susceptibility to infection (e.g., indwelling devices, skin infections, chronic lung diseases); reported risk factors are also sex, with a higher incidence in males; winter months, due to respiratory infections; and recent hospitalizations or residing in long-term care facilities. Sepsis mortality rates are high—up to 50% in cases of septic shock [5,9,10]. Intrahospital mortality has declined from 35% in 2000 to 18% in 2012, resulting in a large number of sepsis survivors with continuous inflammatory changes and immune suppression. These patients suffer from severe persistent impairments and are at an increased risk of death during the following year, recurrent infections, and cardiovascular events [11]. Sepsis and its related conditions represent a major healthcare system burden [12].

There have been several revised definitions of sepsis. The first definitions of sepsis, severe sepsis, and septic shock were released in 1992 [13], with systemic inflammatory response syndrome (SIRS) as its cornerstone. Later, in 2003, a second consensus expanded the list of diagnostic criteria [14]. However, recognizing the limitations of previous definitions, Sepsis-3 was published in 2016, abandoning the term “severe sepsis” and offering The Sequential Organ Failure Assessment (SOFA) and quick Sequential Organ Failure Assessment (qSOFA) scores for organ dysfunction identification and operationalization of clinical criteria. Currently, “sepsis” is defined as life-threatening organ dysfunction caused by a dysregulated host response to infection, and “septic shock” is a subset of sepsis in which the underlying circulatory and cellular/metabolic abnormalities are profound enough to substantially increase mortality [15].

The discussion regarding the best treatment strategies is still ongoing. Early and appropriate administration of antibiotics is recommended. At the moment, protocol-based strategies exist, particularly early goal-directed therapy (EGDT) [16] and guidelines by the Surviving Sepsis Campaign (SSC) [17]. However, evidence for the bundle approach seems to be insufficient, and some meta-analyses fail to show a significant mortality difference from each hour delay of antibiotics [18,19]. And single point-in-time interventions may not be as impactful as initially reported due to the complexity of the pathophysiology of sepsis and resulting organ dysfunction [18,20].

The data on sepsis in Latvia is limited. The reported incidence between 2015 and 2020 was 110–165 per 100,000 population, with an in-hospital mortality rate ranging from 34.7% to 43.8% during the study period. However, the administrative data analysis bears the limitations of the ICD code abstraction approach [21]. The aim of this study was to investigate hospital- and ICU-mortality rates and attempt to characterize sepsis and septic shock cases in an ICU setting in Latvia.

## 2. Materials and Methods

### 2.1. Study Design and Population

In a single-center cohort study, we enrolled 86 patients between December 2014 and February 2018 with community-acquired severe sepsis and septic shock who were admitted to the ICU. Pauls Stradins Clinical University Hospital is a tertiary care facility with a capacity of 890 beds, and its mixed medical and surgical ICU has a capacity of 18 beds (Level 3), with access to invasive monitoring, mechanical ventilation, renal replacement therapy, and extracorporeal membrane oxygenation, and is attended by certified anesthesiologists–reanimatologists. Patients can be admitted to this ICU from the emergency department (ED) or any other hospital department for maintaining or stabilizing vital functions and/or support of ≥2 organ systems (e.g., severe respiratory failure with invasive mechanical ventilation; circulatory shock with medical support, which cannot be provided in the ward; organ replacement therapy, except for chronic dialysis).

### 2.2. Selection of Patients and Data Collection

Eligible cases included ICU-admitted adult patients (age ≥ 18 years) with clinical manifestations of severe sepsis or septic shock upon hospitalization according to the sepsis definitions due date (consensus suggested in the International Sepsis Definitions Conference (Sepsis-2) in 2014–2016 and the Third International Consensus Definitions for Sepsis and Septic Shock (Sepsis-3) after 2016, respectively) [14,15]. Standard definitions of infections in septic patients were used [22]. Community-acquired infection was considered in case of an outpatient setting (hospitalization less than 2 days during the last 90 days) with minimal or no contact with the healthcare system (no long-term intravenous drug administration, invasive devices, such as chronic central venous catheter, urinary catheter, nephrostomy, cystostomy, etc.).

The aim of this study was to focus on community-acquired sepsis. Therefore, we enrolled ICU-admitted patients from the emergency department or another department if referral to the ICU occurred during the first 24 h of hospitalization.

Identification and enrolment of patients were performed by a clinician of the research team based on a review of the medical case history, charts, and other medical documentation of each ICU-admitted patient. Therefore, the evaluation of the inclusion criteria was independent of the initial diagnosis, early sepsis recognition, or administrative data (e.g., diagnosis coding). Demographic data and information on risk factors, clinical characteristics, and diagnostics were collected and analyzed. Information on the clinical presentation upon arrival to the ED and the pre-hospital period was gathered retrospectively.

The intrahospital mortality and initial antibacterial treatment (timeliness and appropriateness) were evaluated. Appropriate empiric therapy was defined according to the initial diagnosis (suspected source of sepsis) and local guidelines. Effectiveness of the treatment was also retrospectively evaluated with regard to an identified organism and in vitro sensitivity to a prescribed antibiotic.

Relevant additional information was gathered during the follow-up period until discharge from the hospital, e.g., established source of infection, identification of causative microorganism, transfer to a general ward, and result of the hospitalization. Patients were excluded from the study if later evidence showed that inclusion criteria were not met (e.g., organ dysfunction due to a non-infectious cause, hospital-acquired infection, or healthcare-associated infection). The data on included patients were revised after the release of Sepsis-3 in 2016 to avoid discrepancies and minimize the study population heterogeneity caused by differences in definitions.

### 2.3. Statistical Analysis

Quantitative data are represented as the mean with standard deviation (SD) and medians with interquartile ranges (IQR), depending on normality. Categorical variables are shown in proportions. Comparisons between groups were made by using the non-parametric Mann–Whitney U test or Fisher’s exact test for categorical values. The statistical significance of the two-tailed *p*-value was set at <0.05. Analyses were performed using Statistical Package for Social Sciences (SPSS) software (IBM SPSS Statistics Version 22, SPSS Inc., Chicago, IL, USA).

## 3. Results

Eighty-six patients were enrolled in this study, including 25 (29%) septic shock cases. However, after the release of the Sepsis-3 guideline, patients included by 2016 were re-evaluated, leaving 23 (27%) cases that met the criteria of septic shock. The majority (90%) was hospitalized by ambulance; 90% (77 cases) were admitted to the ICU from the ED, and the rest of the study population was transferred from other departments during the first 24 h of hospitalization. The patient characteristics and laboratory findings are shown in Table 1. The median door-to-physician time was 5 min (IQR 1–11). A great proportion (77%) of the study population had pre-existing comorbidities, such as a history of chronic heart failure (49%), diabetes (22%), chronic kidney disease (19%), chronic liver disease (9%), chronic obstructive pulmonary disease (8%), malignancies (6%), and immunosupression (5%), with no statistically significant differences between the survivor and non-survivor groups. No significant differences between groups regarding body temperature, C-reactive protein, or pH were recorded.

The median ICU and hospital length of stay was 10 (IQR 3–18) and 17 (IQR 6–28) days, respectively. The length of stay at the hospital was significantly longer for survivors.

**Figure 1:**
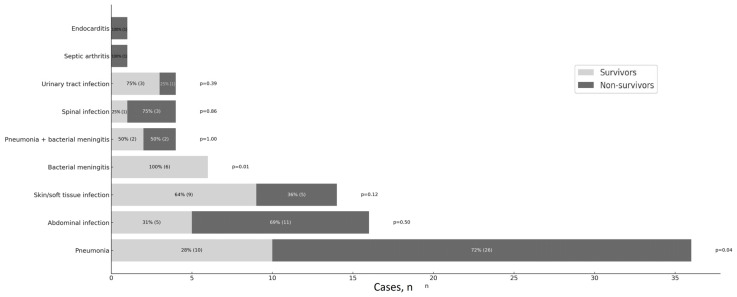
Clinical manifestations—source of infection.

The most prevalent sources of severe sepsis and septic shock were pneumonia (42%) and abdominal (19%) and skin/soft tissue infections (16%), followed by bacterial meningitis (7%), combined bacterial meningitis with pneumonia (5%), spinal infection (5%), urinary tract infection (5%), septic arthritis (1%), and endocarditis (1%) (Figure 1).

**Figure 2:**
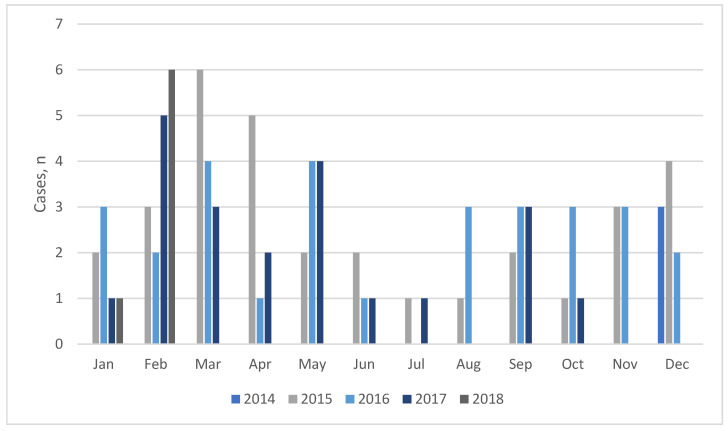
Number of sepsis cases admitted to the ICU per month (2014–2018).

The number of sepsis patients per month is shown in Figure 2. The total count of sepsis/septic shock patients admitted to the ICU ranges from 7–9 cases in the winter months (December–February), 9–13 patients in the spring (March–May), 2–4 cases in the summer (June–August), and 4–9 patients in the autumn (September–November).

The total percentage of the established etiology was 33%. Blood cultures within the first 48 h were taken in 63% of all 86 sepsis cases, but only in 19 of those cases were blood cultures taken before the initiation of antibacterial treatment. And there was no significant difference between the survivor and non-survivor groups (*p* = 0.808) regarding blood sampling prior to the antimicrobials. The positive culture rate was 30% (n = 16). Only one case of multi-resistant microorganism (*Klebsiella pneumoniae* ESBL) was recorded. A causative microorganism was detected in 12 additional cases with alternative methods (Table 2). In five patients, the Legionella urine antigen tested positive.

Appropriate empiric antimicrobial therapy, according to the final diagnosis and local guidelines, was received by 95% of patients, but in three cases, the treatment was retrospectively found to be inappropriate regarding the established etiology. The most commonly initiated empiric antimicrobial treatment was cephalosporins (40%), followed by piperacillin/tazobactam (13%).

Nine patients (10%) received antibiotics within the first hour of hospitalization. The majority (62%) received them in 1 to 6 h, and in 28% of cases, administration occurred in more than 6 h. Administration within the first hour did not significantly impact outcomes (*p* = 0.516). Median door-to-antibiotic time was 225 min (IQR 118–407 min), without significant differences between survivors and non-survivors (*p* = 0.716).

The immediacy of fluid administration was registered. The pre-hospital (ambulance) and ED fluids consisted of crystalloids. In the ICU, colloids were also administered. Forty patients (47%) received fluids in the ambulance; another 27 patients (31%) received them during the first hour in the ED. No statistically significant differences between the survivor and non-survivor groups were detected.

The overall hospital and ICU mortality rates were 58% and 45%, respectively. There were four (5%) deaths during the first 24 h. In the logistic regression analysis (Table 3), age was independently associated with higher odds of mortality (OR 1.07, 95% CI 1.03–1.24, *p* = 0.010).

## 4. Discussion

Although the sample size is rather small, this study provides unique insight into the real-life situation regarding sepsis morbidity, since the inclusion process was not dependent on diagnostic codes or other administrative data. Due to prospective case-by-case enrolment based on clinical assessment, we were able to identify and exclude false-positive cases because codes for organ dysfunction would not necessarily originate from infection, and, on the contrary, reliance on the ambulance or ED physicians’ ability to recognize sepsis was eliminated. It has also been stated in the literature that chart-based clinical validation of sepsis cases identified through administrative databases shows a severalfold higher incidence rate [5]. However, several concerns persist regarding patient selection. Enrolment of cases took place in the general ICU. Therefore, the possible influences of ED hospitalization policies, ICU capacity, and availability are a matter of discussion, as a proportion of septic patients with certain infections are usually admitted to other ICUs in the same hospital or referred to specialized centers (e.g., bone infections). For example, a septic patient with severe pneumonia and respiratory failure can also be admitted to a PSCUH pulmonological high-dependency unit, where non-invasive ventilation is available; or septic endocarditis cases with acute cardiologic emergency can be hospitalized in a cardiological ICU. The cardiac–surgical ICU in the PSCUH only serves patients after cardiac surgery and major blood vessel reconstructions. Therefore, it is a less-likely department for community-acquired sepsis patients to be admitted to. A subgroup of enrolled patients was admitted to the ICU after emergent or urgent surgery. Particularly in cases of abdominal infections, the evaluation of sepsis is complicated, since organ dysfunction may not be attributable to infection alone. The heterogeneity of the study population has been widely emphasized as an inherent limitation to the nature of sepsis research.

The number of patients enrolled indicates that sepsis cases are underreported on a national level. According to the Latvian Centre for Disease Prevention and Control, there were only 45 and 54 reported fatal sepsis cases (2.3 and 3.0 per 100,000 population) annually in 2016 and 2018, respectively, with the numbers slightly rising during the recent years, reaching 5.2 and 8.8 per 100,000 population in 2019 and 2022, respectively [23,24]. The official data on sepsis incidence in Latvia is not available. However, the rates are well below the results published from other countries [3]. Knoop et al. reported that the incidence of sepsis cases was 140/100,000 inhabitants in Norway, with a hospital mortality of 19.4% (27.16/100,000 inhabitants) [10].

We report intrahospital and ICU mortality rates of 58% and 45%, respectively, in ICU-admitted patients with community-acquired severe sepsis and septic shock, which are higher than in other studies. Reported intrahospital mortality in Sweden was 29% [25]. ICU and intrahospital mortality in Finland were 16% and 28%, respectively [26]. Septic shock patients were studied in France, where ICU, day 28, and hospital mortality rates were 39.5%, 42%, and 48.7%, respectively [9]. However, the intrahospital case fatality rate in Latvian administrative data ranged from 34.7% in 2015 to 41.6% in 2018 [21]. In our study, the patients in the non-survivor group were significantly older, and the association between age and survival of a critical illness is well-established [6,27]. In addition, the limited capacity of the ICU would mean that the most severe and complicated cases, mostly on multiple organ support, would be admitted there. Overall, the number of ICU beds per capita in Latvia (14.9 per 100,000 population in 2021) is lower than in many other European countries (e.g., 45.5, 29.3, and 27.8 per 100,000 population in the Czech Republic, Germany, and France, respectively) [28]. Delayed seeking of medical help and hospitalization of septic patients is another possible factor for high mortality that needs to receive proper attention in further research, since public awareness of early sepsis recognition is low, according to initial explorations [29]. In addition, there is no legislation on the do-not-resuscitate order in Latvia. Therefore, analyzing the doctors’ perceptions on obligations to treat and treatment escalation decisions (including ICU admission) in cases of severe comorbid conditions would probably add valuable insights and understanding to the results of this study. Critically ill patients mostly present with altered mentation, and data on comorbidities is often missing due to limited or nonexistent access to previous health records. Also, the distinction between community-acquired and healthcare-associated sepsis is occasionally rather complicated. Moreover, a further sub-analysis of patient pathways after discharge from the ICU (e.g., readmitting from regular wards) would add to a better understanding of the outcomes. SOFA, SAPS II, and APACHE scores were not available for this study, although the data would allow us to compare our patient population with other studies in order to assess the possible contributing factors to the mortality rates found in this study.

The majority of studies report pneumonia and abdominal infections as the most common sites of infection, and we had similar findings regarding sepsis source, followed by skin and soft-tissue infections [30,31]. The predominance of Gram-positive bacteria was observed, which is consistent with pneumonia being the most prevalent source of infection. The positive culture rate in this study was around one-third, which is consistent with other reports [31,32]. However, a significant portion (65%) of blood cultures were obtained after the initiation of antimicrobials. Only one case of an ESBL-positive microorganism was recorded; no other multi-resistant strains were found in our patient group with community-acquired infection as a source of sepsis. Five patients tested positive for *Legionella pneumophila* infection, which is a reportable finding since Legionnaires’ disease is a subject of international surveillance.

Regardless of the small sample size, seasonality was observed in the number of sepsis cases admitted to the ICU. In other reports, severe sepsis was more frequent during the cold autumn and winter months [27,31]. However, in this study, we detected fewer admissions with sepsis only during the summer months.

The cornerstone of sepsis treatment recommended by the Surviving Sepsis Campaign guidelines, which are widely utilized in Latvia, is the timely administration of adequate broad-spectrum antibiotics, along with circulatory support with fluids and vasopressors [17]. The majority of patients (95%) received appropriate empiric antimicrobial treatment. However, defining the ‘‘appropriateness’’ of antibiotics can be challenging, as it is ultimately based on identifying a causative pathogen. In our study, in 3% of cases, the antimicrobials chosen according to the initial diagnosis (site of infection) and local guidelines were inappropriate regarding the established etiology. The approach to evaluation of antibacterial treatment adequacy in other studies and reports is inconsistent, and no widely accepted definitions exist regarding the timeframe for combination therapy administration, although some reports define treatment as appropriate if at least one effective antibiotic is initiated, while research on the efficacy of combination antimicrobial therapies is still ongoing [33,34].

Timely administration of antibiotics is reported to be an important prognostic factor [35]. The median door-to-antibiotic time in our study was 225 min (IQR 118–407), compared to other studies where antibacterial treatment was initiated in 86 min [25] to 3 h and more [19]. Although the difference was not statistically significant, antibiotic administration in the survivor group was 280 min (IQR 126–456) from the moment of hospitalization, as compared to 212 min (IQR 111–404) in the non-survivor group. However, this could be due to confounding by indication (patients in more serious conditions receive treatment sooner). Overall, 10% of patients received antibiotics in the first hour. Furthermore, it should be emphasized that the reference point in this case is admission time, since no data on sepsis recognition timing exists. Meanwhile, the median door-to-physician time was 5 min (IQR 1–11), which was shorter than in the other reports [25], likely due to the prioritization of severely ill patients at the ED via the patient triage process.

Community-acquired sepsis in Latvia shares the same low-resistance setting as Scandinavia, and although Latvia is a high-income country according to the World Bank, regional healthcare approaches, access to healthcare, and sociopsychological aspects differ. Preventive measures, such as the prophylaxis of infections, vaccination, management of chronic diseases, and treating sepsis early, should receive greater focus from healthcare workers, patients, and health policy makers. Further in-depth studies are needed, not only national registry and data pooling from other hospitals, but also qualitative and behavioral studies should be encouraged. Raising sepsis awareness among healthcare professionals is crucial to improving outcomes.

## 5. Conclusions

The number of patients enrolled indicates that sepsis cases are underreported on a national level. The results also demonstrate high mortality rates among ICU patients with community-acquired severe sepsis and septic shock, as compared to other reports. The initial analysis indicates that the early sepsis signs and, in particular, organ failure are overlooked. Interventions based on the latest sepsis definitions have been initiated, with SOFA and qSOFA scores integrated into local sepsis guidelines to facilitate the early recognition of sepsis and organ failure, thus improving treatment outcomes.

## Figures and Tables

**Table 1:** Patient characteristics and instrumental findings.

Variable	Septic Cohort, n = 86	Survivors, n = 36	Non-Survivors, n = 50	*p*-Value
Male sex, n (%)	51 (59.3)	26 (72.2)	25 (50.0)	0.084
Age, median (IQR)	65 (52–77)	57 (46–68)	69 (56–79)	<0.001
ICU *—admitted from ED ^, n (%)	77 (89.5)	28 (77.8)	49 (98.0)	0.080
Septic shock, n (%)	23 (26.7)	6 (16.7)	17 (34.0)	0.176
qSOFA † ≥2 points, n (%)	28 (32.6)	8 (22.2)	20 (40.0)	0.219
Leucocytosis, n (%)	52 (60.6)	27 (75.0)	25 (50.0)	0.255
Leukopenia, n (%)	11 (12.7)	1 (2.8)	10 (20.0)	0.034
Lactate—mmol/L, median (IQR)	2.5 (1.7–4.0)	1.8 (1.4–2.7)	2.9 (2.3–4.7)	0.149
pH, median (IQR)	7.32 (7.24–7.38)	7.34 (7.24–7.41)	7.31 (7.24–7.37)	0.457
CRP—mg/L, median (IQR)	209 (130–299)	199 (130–288)	213 (128–316)	0.720
Door-to-antibiotic time—minutes, median (IQR)	225 (118–407)	280 (126–456)	212 (111–404)	0.716
ICU stay—days, median (IQR)	10 (3–18)	11 (7–17)	7 (2–22)	0.150
Length of hospital stay—days, median (IQR)	17 (6–28)	25 (17–35)	8 (3–24)	<0.001

* ICU—intensive care unit; ^ ED—emergency department; † qSOFA—quick Sequential [Sepsis-Related] Organ Failure Assessment Score.

**Table 2:** Microbiological findings of blood cultures and alternative identification methods.

Organism	Cases, n = 28
*Streptococcus pneumoniae* (Culture or PCR ** on CSF ^ or urinary antigen)	9
*Staphylococcus aureus* (MSSA *)	6
*Legionella pneumophila* (urinary antigen)	5
*Klebsiella pneumoniae*	3
*Escherichia coli*	2
B group streptococci (*Streptococcus agalactiae*)	1
A group streptococci (wound swab)	1
*Neisseria meningitidis* (PCR on CSF ^)	1

* Methicillin-susceptible *Staphylococcus aureus*; ** PCR—polymerase chain reaction; ^ CSF—cerebrospinal fluid.

**Table 3:** Logistic regression analysis of risk factors associated with sepsis mortality.

Variable	Odds Ratio (OR)	95% CI	*p*-Value
Age	1.07	1.03–1.24	0.010
Male gender	0.81	0.20–3.32	0.774
Septic shock	1.41	0.30–6.57	0.663
CRP	1.00	0.96–1.01	0.981
Lactate	0.96	0.75–1.22	0.717
Door-to-antibiotics time	1.00	1.00–1.01	0.864
Chronic heart failure	0.53	0.13–2.17	0.375
Diabetes	1.26	0.58–2.71	0.563
Chronic kidney disease	0.34	0.13–1.21	0.082
Chronic liver disease	1.16	0.51–2.46	0.786
Chronic obstructive pulmonary disease	2.46	0.84–7.22	0.101
Malignancies	0.08	0.01–1.20	0.068

## Data Availability

Data are contained within the article.
